# U(VI) removal from diluted aqueous systems by sorption–flotation

**DOI:** 10.1038/s41598-022-19002-0

**Published:** 2022-10-10

**Authors:** Carolina Constantin, Ioana-Carmen Popescu, Ovidiu Oprea, Ligia Stoica

**Affiliations:** 1 Department of Inorganic Chemistry, Physical-Chemistry and Electrochemistry, Faculty of Applied Chemistry and Materials Science, University “POLITEHNICA” of Bucharest, 313 Splaiul Independentei, 060042 Bucharest 6, Romania; 2 Research and Development National Institute for Metals and Radioactive Resources, INCDMRR-ICPMRR, Laboratory of Environment Protection Technics and Technologies, 70 Blvd. Carol I, 020917 Bucharest 2, Romania

**Keywords:** Chemical engineering, Environmental chemistry, Pollution remediation, Environmental sciences, Chemistry

## Abstract

The legacies of past uranium mining and milling activities for nuclear fuel fabrication continue to be a cause of concern and require assessment and remedial action for researchers worldwide. The discharge of uranium contaminated water into the environment is a matter of regulation (World Health Organization, WHO—15 μg/L, Romanian Legislation, RO—21 μg/L), environment and health. Therefore, various removal technologies of U(VI) from diluted aqueous solutions include chemical precipitation, ion exchange, adsorption, immobilization on zero-valent iron nanoparticles, etc. have been extensively applied. Our previous research has studied the removal of U(VI) from diluted aqueous systems such as mine waters using Fe^0^-based nanomaterials synthesized in the laboratory (NMS) (Crane et al. in Water Res 45:2391–2942, 2011). The carbonate rich aqueous system was treated with NMS to remove U(VI). It was observed that after half an hour of reacting time only about 50% was removed due to its high tendency to form stable soluble carbonated complexes. Considering that, the present article aims to investigate the Sorption/Flotation technique, by using a sorbent generated in situ Fe_2_O_3_· nH_2_O and sodium oleate surfactant to remove U(VI) from diluted aqueous systems and to update the knowledge on the mechanism of process. In order to determine the removal efficiency of U(VI), the influencing factors were studied: pH, sorbent dose, surfactant concentration, contact time, stirring rate, the U(VI) concentration, air pressure in pressurized water recipient, and the effect of some accompanying heavy metals ions (Cu(II), Cr(VI), and Mo(VI)). The removal efficiency (%R) was monitored and its maximum values allowed to establish the optimal separation parameters (the established process parameters), which were validated on real mine water samples (MW). High U (VI) removal efficiencies %R > 98% were obtained. The Sorption/ Flotation technique was applied to remove U(VI) from two types of real mine water samples, namely ”simple” and ”pre-treated with NMS”, respectively. For the mine water samples pre-treated with NMS, it worked in two variants: with and without pH correction. For pH range = 7.5–9.5, molar ratios [U(VI)] : [Fe(III)] = 1 : 75, [U(VI)] : [NaOL] = 1 : 1 × 10^–2^, contact time 30 min., stirring speed 250 RPM, initial concentration of U(VI) 10 mg·L^−1^, air pressure in pressurized water recipient *p* = 4 × 10^5^ N·m^−2^ is obtained %R > 98%. It has been found that Sorption / Flotation can function with good %R values as a stand—alone operation or in tandem with NMS pre-treatment of mine water and pH adjustment proved to be highly efficiency (C_U(VI)_ < 1·10^–3^ mg·L^−1^).

## Introduction

Radioactive pollution of the environment caused by uranium ores hydrometallurgical processing, in addition to the cross-contamination generated by other heavy metals used in this industry, is still a challenge for scientists, and a major threat to human health worldwide^2–4^. Mine water generated by the weather events is an important radioactive pollutant and mobilizes significant amounts of U(VI), in addition to other accompanying heavy metals such as Cu (II), Cr (III + VI), and Mo (VI), and consequently, needs highly efficient remediation technologies^3,5^. Unfortunately, the developed remediation technologies such as complexing processes^6^, co-precipitation^7–10^, redox reactions^9^, ion exchange^11,12^, solvent extraction^13,14^, adsorption on different materials^15–19^ bioremediation^20,21^ and immobilization on nanomaterials^5,18,22–26^ presents specific advantages and disadvantages. One example for specific advantage is the develop of new sorbents with changed properties that offer a multitude of improved applications, including selectivity. In case of removal of uranium from aqueous solution some research can be noticed for this purpose. Chitosan cross-linked using glutaraldehyde in the presence of magnetite. The resin was chemically modified through the reaction with tetraethylenpentamine (TEPA) to produce amine bearing chitosan. This resin showed a higher affinity towards the uptake of UO_2_^2+^ ions from aqueous medium^27^. Schiff’s base chitosan composite with magnetic properties. This composition showed high affinity and fast kinetic for the sorption of UO_2_^2+^ ions^28^. Magnetic chitosan nanoparticles functionalised by grafting diethylenetriamine (DETA) and dithizone for improving U(VI) sorption at pH around 5^29^. The phosphorylation of guar gums combined with chitosan preparing an efficient sorbent for the removal of U(VI) from slightly acid solutions. In addition if it is done phosphorylation of guar gums/magnetite/chitosan nanocomposites has antibacterial effects against both Gram+ and Gram− bacteria^30^. Another interesting new sorbent for U(VI) are silica beads functionalized with urea or thiourea-based polymers^31^. Examples for disadvantages are the chemical methods, ion exchange, and solvent extraction. There are highly efficiency in treating effluents that contain large amounts of pollutants, but are prohibitive in remedying diluted aqueous systems (10^–3^–10^–6^ M solutions).

Flotation is one of the absorptive bubble separation techniques, which involves the removal of surface inactive ions from homogeneous and heterogeneous aqueous systems by the introduction of a surfactant to become surface-active ions and subsequent passage of gas micro disperse bubbles through the solution in a foam separation column. The surface-active ions, which are absorbed on the surfaces of the rising bubbles, can be carried upward to the top of the foam separation column, and thus removed from the aqueous system as condensed foam (sublate).

In the separation process the properties of participant phases are important: superficial interface properties of liquid phase; high hydrophobia and low density for species in foam concentrated; homogeneous dimension of gas bubbles, which provides the mass transfer liquid-foam; the optimum gas flow for the bubble-particles aggregation in foam.

The different technological variants such as ion flotation^4,32–35^, precipitate flotation^36^, sorption–flotation^12,35,37–40^, colloidal adsorbing flotation^41^, electro-flotation^42^, flotoextraction^43,44^, have proved their highly decontamination efficiency of a wide variety of diluted aqueous systems^45–52^.

The main advantages are high selectivity, adaptability, high removal efficiency, possibility of being applied for the removal of ionic, molecular, colloidal, and micro-dispersed species of inorganic or organic nature^35,48–52^. However, some of them have disadvantages, namely electro-flotation, which consumes energy, and the flotation with dispersed gas, which provides non-homogenous bubbles and requires high quality and resistant porous material. Among the bubble generation techniques, the Dissolved Air Flotation (DAF) technique is preferred because it provides small homogeneous bubbles in situ.

DAF application involves two variants: (a) direct pressurization by introducing the airflow into the water sample conditioned with reagents subjected to flotation; (b) dilution with recirculated water under pressure of the sample conditioned with reagents, before flotation. Industrial is the last option because it is cost-effective and works in small, compact, and relatively simple installations. Rapidity (flotation contact time is less than 5 min.), versatility (removal of organic substances and heavy metals such as copper, chromium and molybdenum), simplicity of installation, are other advantages that recommend this method of separation.

The mobility of U(VI) is largely dependent on changes in pH, variation of redox potential in the environment, and the presence of other neutral and/or ionic species, such as humic acids, sulfate, phosphate, and carbonate ions, which interact with the uranyl ions, and turns them into highly soluble complexes. For example, carbonate generates highly stable uranyl-carbonate complexes and plays a key role in its biogeochemistry and bioavailability^53^.

Considering the complex chemistry of uranium-contaminated mine waters, in addition to environmental pollution caused by other industries using toxic heavy metals such as Cu (II), Cr (VI), and Mo (VI), this study aims to investigate the removal of U(VI) by Sorption / Precipitate flotation from mono- and multi-contaminated aqueous systems as natural analogues, and, respectively, real mine water samples, in order to update the acquired knowledge on the process mechanism.

The sorbent used in this study is generated in situ consists of Fe_2_O_3_ × n H_2_O and was selected considering iron’s physicochemical properties and high separation efficiency of a wide variety of contaminants, including U(VI) and the accompanying heavy metals^5,23,24,54^. This choice was made to obtain the advantages offered by: (i) the circulation of a small volume of reagent with Fe(III) to generate the adsorbent support; (ii) the reduction of the costs of adsorbent support obtaining; (iii) ensuring the optimal contact with U(VI) through its loose and flaky structure; (iv) the reduction of the reaction time and the volume of waste generated. Although iron hydroxide is not unique, it is an environmentally friendly and low cost alternative to synthesized sorbents.

In order to increase the hydrophobicity of the sorbent loaded with U(VI), a collector (surfactant) was introduced into the system, which in this case is sodium oleate (NaOL), C_18_H_33_O_2_Na. It has been preferred over others because it is a common and inexpensive reagent due to its low toxicity, accessibility, high availability, and proven safety in its food uses as a binder, emulsifier, anticaking agent, and indirect additive^55^. The long C-chain of sodium oleate explains the high hydrophobicity and surface-active (surfactant) properties of sodium oleate^48–52^.

The research presented in this paper is justified by the practical scientific interest shown above^4,12,32–35,37–39^.

Previous research^1^ on the removal of U(VI) on Fe^0^-based nanomaterials synthesized in the laboratory (NMS) have proved their efficiency in U(VI) removal from carbonate-rich mine water in about one hour, but after more than 24 h of reaction time a desorption process due to the soluble appearance of uranyl-carbonate complexes^5^. Thus, the combined NMS—Sorption/Flotation tandem technology is becoming a promising treatment alternative. Therefore, the novelty of this paper compared to previous research is the proposal of a new remedial technology that uses the reactivity of iron-based nanomaterials and separation efficiency of DAF technique.

## Materials and method

### Reagents

All the reagents were MERCK analytical grade. All solutions were prepared using Mili-Q purified water (resistivity > 18.2 MΩ cm).

- Na_4_[UO_2_(CO_3_)_3_], stock solution (1 g·L^−1^ U(VI)), and work solution (10 mg·L^−1^) were prepared using uranyl acetate (UO_2_(CH_3_COO)_2_·H_2_O) and anhydrous sodium carbonate (Na_2_CO_3_);
- Cu (NO_3_)_2_, MoO_3_·H_2_O, and K2CrO_4_, work solutions (10 mg L^−1^) of Cu(II), Cr(VI), and Mo(VI);
- NaOH and HCl, pH adjustment solutions (0.01 M and 0.1 M);
- FeCl_3_, solutions (0.01 M and 0.1 M) for sorbent in situ generated;
- NaOL solutions: 0.25 M, 0.25 × 10^–3^ M and 0.025 × 10^–3^ M.
- NMS (Fe^0^-based nanomaterials) synthesized in the laboratory^1^

### Equipment

- Heidolph Vibramax 100 stirrer, with variable speed.
- 290A ORION pH-meter;
- UNICAM PAY SP9 atomic absorption spectrophotometer for Cu (II), Cr (VI), and Fe (III) determination;
- CINTRA 404 UV–VIS spectrophotometer for U(VI) and Mo (VI) determination.
- UHPLC PLATINblue for NaOL determination
- FT-NIR spectrophotometer MB3600-AAA for IR spectra.
- Netzsch analyzer TG 449 C STA Jupiter for solid samples thermal analysis.

### Experimental method

#### Sorption/flotation experiments with sorbent generated in situ

The experiments were performed in batch mode. The U(VI) sample (200 mL) was contacted with Fe (III) solution mixed with NaOH 0.1 M at various molar ratios [U(VI)]: [Fe (III)] stirred continuously for a previously set time of 30 min^56^. After adjusting the pH, the sample was contacted with the surfactant, (NaOL) in various molar ratios [NaOL]: [U(VI)] and transferred to the flotation cell, which is coupled to a pressured water recipient. The recipient is filled with water saturated with air at a pressure of 5 × 10^5^ N·m^−2^. An aliquot of water under pressure (dilution ratio V_sample_: V_water_ = 3 : 1) was introduced into the base of the flotation cell and uniform-sized microbubbles were generated. Thus, they adhered to the surface of the formed solid (sorbent generated in situ and U(VI) loaded on its surface) and it rises to the top of flotation cell. The flotation time was 5 min until all foam was separated at the top. The residual concentration of U(VI), Fe (III), and NaOL was analyzed. All experiments were triplicated.

The study of influencing factors (pH, molar ratio, metallic ion concentrations, necessary air, etc.) by %R = f(property)_max_ established the optimum conditions for U(VI) separation.

The removal efficiency was calculated according to the equation:

1 $$ \% {\text{R }} = \, \left[ {\left( {{\text{C}}_{{\text{i}}} - {\text{ C}}_{{\text{f}}} } \right)/{\text{C}}_{{\text{i}}} } \right] \, \times {1}00, $$

where,

- C_i_ is the initial concentration of metallic ions(mg·L^−1^);
- C_f_ is the final concentration of metallic ions (mg·L^−1^).

The adsorption process of U(VI) under working conditions is characterized by an isothermal dynamics and the kinetic models, respectively. The obtained results^56^ suggest that the process is mixed and involves both physical and chemical interactions between U(VI) and Fe(III) aqueous species (co-precipitation). Based on the corelation coefficients (R2), the sorption equilibrium data fitted to the isotherm models in the following order: Langmuir (0.9808) > Temkin (0.8715) > Freundlich (0.8344). The close values of KF, Qe_exp_ and Qe_calc_ suggest that the process involve chemisorption in good agreement with the fact that it has followed the pseudo-second order kinetics as confirmed by other studies^57,58^.

#### Optimal parameters’ validation experiments

Two types of mine water samples (MW), namely “simple”and “pre-treated with NMS” respectively (V_sample_ = 400 mL, pH range = 7.5–9.5, m_NMS_ = 0.1 g, τ_contact_ = 30 min, stirring rate 250 RPM) were subjected to previously studied Sorption/Flotation process. Mine water samples (MW1–3) were collected from a former uranium mining site situated in the Banat region.

Regarding the ”pre-treatment with NMS” samples, it is mentioned that the nanomaterial used has the following characteristics^1^:

- the surface area (by BET analysis) was 14.8 m^2^/g for over 80% of the studied nanoparticles;
- particle size distribution (by TEM analysis) in the range 0–50 nm;
- XRD analysis of crystallinity revealed disordered / amorphous structure;
- XPS analysis of chemical composition of the surface led to % Fe = 30.5, % O = 32.1, % C = 14.5 and % B = 22.9;
- oxide thickness (by XPS analysis) was 3–4 nm;
- surface chemistry (Fe^0^ / Fe^2+^  = 0.02 and Fe^2+^ / Fe^3+^  = 0.38) ^1^;

## Results and discussions

### Influencing factors

#### Flotation pH

**Figure 1:**
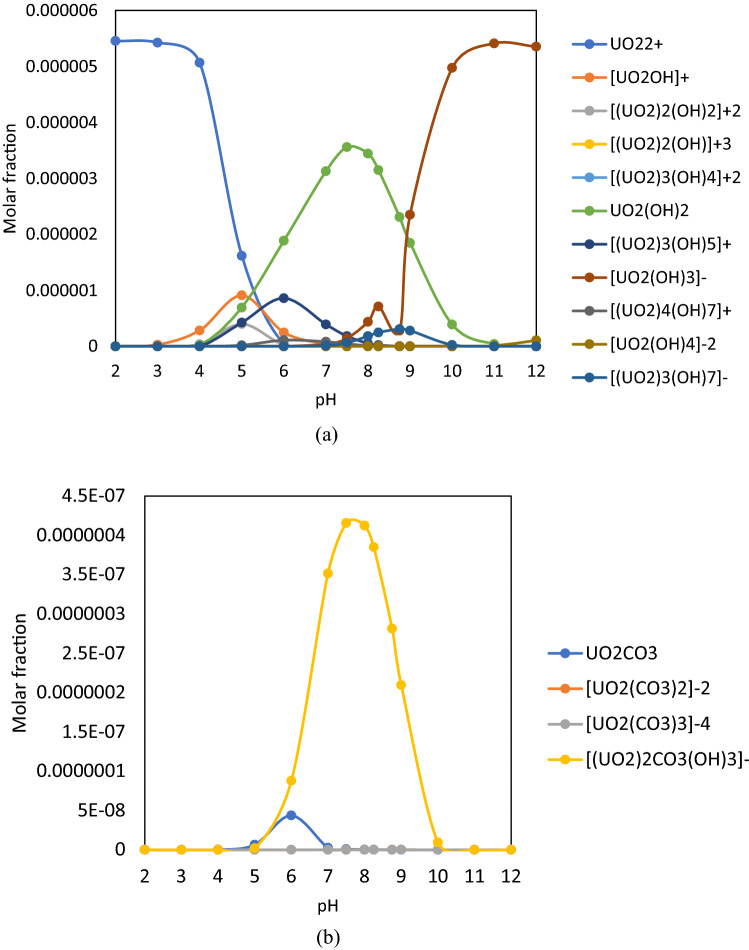
U(VI) species in the mixture U(VI) + Fe(III) calculated by Phreeqc Interactive 3.2.2 software and llnl.dat database. (**a**) hydroxide- complexes; (**b**)—carbonatic-complexes.

The pH is extremely important because it determines the charge, the structure, and the concentration of U(VI) species in dilute aqueous systems (Fig. 1). The U(VI) species were calculated using Phreeqc Interactive 3.2.2 software and llnl.dat database considering only the simple aqueous solution of Na_4_[UO_2_(CO_3_)_3_] containing 0.042 mM U(VI) (10 mg·L^−1^ U(VI)), respectively (main concentration of experimental samples). The pH ranged from 2 to 12 to cover all the types of natural waters, such as highly acidic ones from acid mining drainage and those from the uranium mining industry. The ionic strength was determined by the software.

Species calculations were performed taking into account the simple aqueous system containing only the simple chemical substance without any addition of salts to ensure a constant ionic strength. Ionic strength was calculated by the software. The sum of molar fractions was 1, considering all the species involved. Species with very small molar fractions were not displayed.

The curves obtained for [U(VI)] = 0.042 mM are in agreement with literature^59^. According to the calculated data displayed in Fig. 1, the probable U(VI) species occurring in the pH range 7.0–9.5 are: (a) hydroxide complexes—UO_2_(OH)_2_, [UO_2_(OH)_3_]^−^, [(UO_2_)_3_(OH)_7_]^−^, and carbonate complexes—UO_2_CO_3_, [UO_2_(CO_3_)_2_]^−2^, [UO_2_(CO_3_)_3_]^−4^ and [(UO_2_)_2_CO_3_(OH)_3_]^−^ in agreement with literature^53,59^. The U(VI) hydroxide—and carbonate species were separately plotted, due to the different fraction ratios.

**Figure 2:**
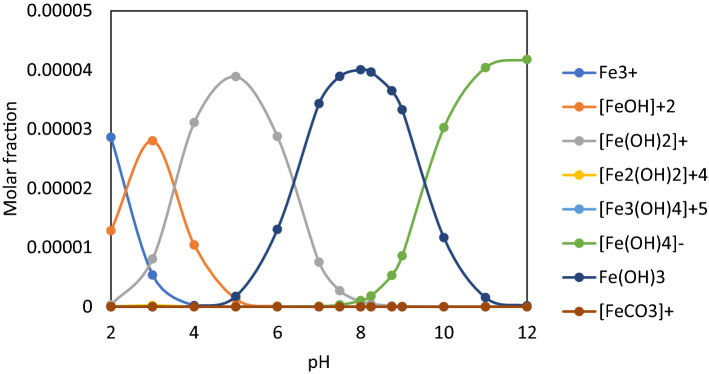
Fe (III) aqueous species in the mixture U(VI) + Fe(III) calculated by Phreecq Interactive 3.1.1-8288 software using llanl.dat database.

Figure 2 were showed the sorbent Fe(III) species calculated by Phreeqc Interactive 3.2.2 software and llnl.dat database.

It is observed from Fig. 2 that Fe_2_O_3_·nH_2_O is formed in the pH range between 7.0–9.0, identical to that of [(UO_2_)_2_CO_3_(OH)_3_]^−^ and UO_2_(OH)_2_. Therefore, as a result, there is a competition between these species. Their formation respects the ascending order of solubility product (K_sp_) K_sp, Fe(OH)3_ = 4 · 10^–38^ < K_sp, UO2(OH)2_ = 1.1 · 10^–22^ < K_sp, UO2CO3_ = 1.8 · 10^–12^ < K_sp, FeCO3_ = 10^–10.5^^60,61^.

**Figure 3:**
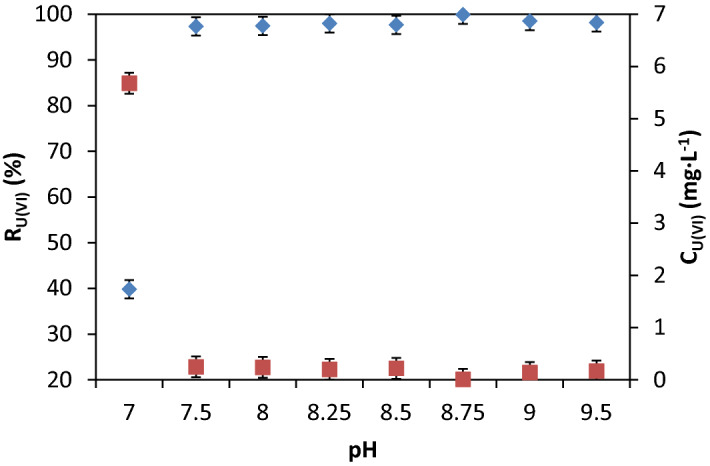
%R_U(VI)_ and C_U(VI)_ = f(pH), V_sample_ = 200 mL, stirring rate 250 RPM, [U(VI] : [Fe (III)] : [NaOL] = 1 : 100 : 1, contact time 30 min, p = 4 × 10^5^ N·m^−2^, dilution ratio V_sample_ : V_water_ = 3 : 1

The influence of pH on removal efficiency has been studied on sorption / precipitate flotation by the function %R = f(pH) (Fig. 3).

**Figure 4:**
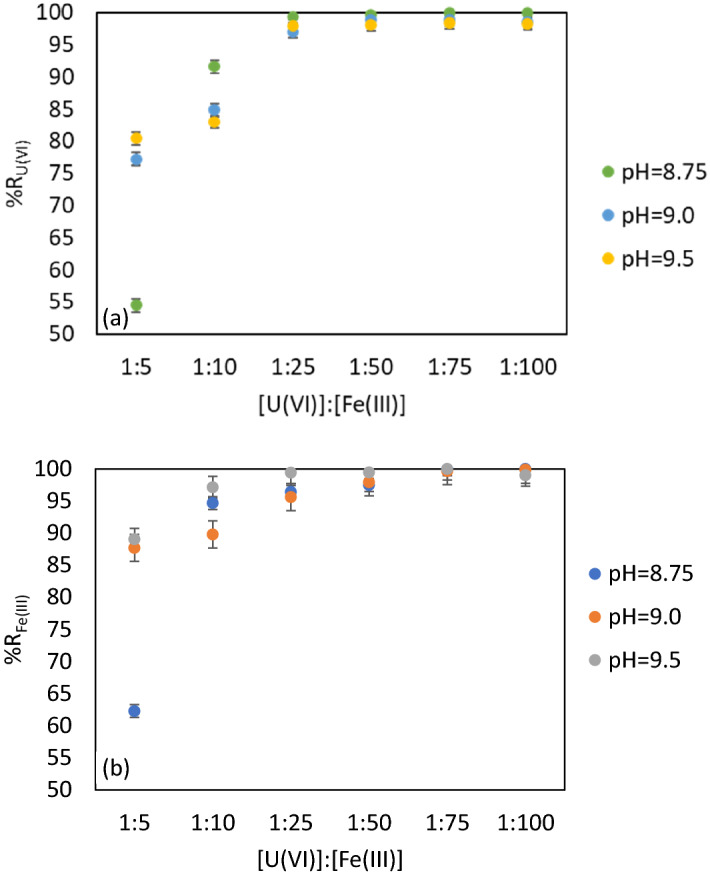
(**a**) %R_U(VI)_ = f ([U(VI)] : [Fe(III)]) in the optimal pH range; (**b**) %R_Fe(III)_ = f([U(VI)] : [Fe(III)]) in the optimal pH range (V_sample_ = 200 mL, stirring rate 250 RPM, contact time 30 min, *p* = 4 × 10^5^ N·m^−2^, dilution ratio V_sample_ : V_water_ = 3:1).

The U(VI) samples (200 mL) of 10 mg·L^−1^ U(VI) were contacted with Fe (III) solution at molar ratio [U(VI)]: [Fe (III)] = 1: 100, which was determined by preliminary tests^62^ under constant stirring (250 RPM) for 30 min to generate the sorbent in situ (Fe_2_O_3_·nH_2_O). The pH adjustment was performed in the pH range 7.0–9.5 corresponding to the maximum sorbent amount (Fig. 4). After adjusting the pH, the sample was contacted with the surfactant (NaOL) at the molar ratio [NaOL]: [U(VI)] = 1:1^51,63^, transferred to the flotation cell and diluted in a dilution ratio V_sample_: V_water_ : = 3 : 1 with distilled water under pressure, as described above. Residual concentrations of U(VI) were analysed.

The best U(VI) removal efficiencies (%R > 98%) very close in values were obtained at pH range 7.5–9.5, which may be explained by the physicochemical interactions of U(VI) species studied by sorption and/or precipitation with sorbent species generated in situ: [Fe (OH)_2_] ^+^, Fe (OH)_3_ and [Fe (OH)_4_]^−^ plotted in Fig. 2. Wang et al.^64^ have demonstrated that the sorbent’s surface charge is influenced by aging^65^ by its concentration and the zeta potential of the sorbents generated in situ is positive at pH around 8, then becomes negative^66^.

#### U(VI) : sorbent dose, [U(VI)]: [Fe(III)]

The sorbent dose is important for the highly efficient removal of U(VI) species from diluted aqueous systems by sorption / flotation, the possible interactions being physical (sorption) or chemical (co-precipitation). The optimum amount of sorbent is a minimum of solid waste, but a maximum of adsorbent support that ensures maximum efficiency.

The different molar ratios [U(VI)]: [Fe(III)] ranging between 1: 5 and 1: 100 were provided using known volumes of 0.1 M and 0.01 M FeCl_3_ solutions. The pH adjustments in the range 7.5–9.5 were made using 0.1 M and 0.01 M NaOH solutions. The studies were performed for the pH values 8.5, 9.0, and 9.5 (pH of real mine waters). Surfactant’s concentration used was the same for all these experiments to provide the best solid phase separation. Figure 4a, b show the obtained results for the residual concentrations of U(VI) and Fe (III) and the recovery efficiency.

Lower molar ratios [U(VI)] : [Fe(III)] between 1:5 and 1:25 were not adequate because U(VI) concentrations exceed the legal limit at the international level^67^. The sorbent—contaminant contact surface was not efficient for the removal of U(VI) according to the legislation in force.

The molar ratio [U(VI)]: [Fe (III)] = 1:75 and pH = 8.75, 9.0 and 9.5 corresponds to a maximum efficiency of U(VI) and Fe(III) removal, %R = 99.96% (C_U(VI)_ = 0.0044 mg·L^−1^ and C_Fe(III)_ = 0.01 mg·L^−1^ as mean value).

#### Molar ratio, [U(VI)]: [NaOL]

In the precipitate flotation, the surfactant consumption is substoichiometric molar ratio. However, the concentration is important because floatability should increase in terms of concentrations below the critical micellar concentration of the surfactant^68^.

To provide the best separation of the sorbent loaded with U(VI), it is necessary to determine the optimal amount of NaOL, which increases the solid phase’s hydrophobicity and floatability due to its long C-chain^63^. Aqueous sodium oleate species are pH-dependent, therefore the same pH values were provided to run the experiments.

According to^51,63^ the chemical equilibria that should be considered between the oleate species are:

2 $$ {\text{RH}}_{s} \mathop \leftrightarrow \limits^{{K_{1} }} {\text{RH}}_{aq} \quad {\text{pK}}_{{\mathbf{1}}} = {7}.{6}0 $$

3 $$ {\text{RH}}_{{{\text{aq}}}} \mathop \leftrightarrow \limits^{{{\mathbf{K}}_{2} }} {\text{R}}^{ - } + {\text{H}}^{ + } \quad {\text{K}}_{{2}} = {4}.{95} $$

4 $$ {\text{RH}}_{{{\text{aq}}}} + {\text{R}}^{ - } \mathop \leftrightarrow \limits^{{{\text{K}}_{3} }} {\text{R}}_{2} {\text{H}}^{ - } \quad {\text{pK}}_{{3}} = - {4}.{95} $$

5 $$ 2{\text{R}}^{ - } \mathop \leftrightarrow \limits^{{{\text{K}}_{4} }} {\text{R}}_{2}^{2 - } \quad {\text{pK}}_{{4}} = - {4}.00 $$

6 $$ {\text{R}}_{2} {\text{H}}^{ - } + {\text{Na}}^{ + } \mathop \leftrightarrow \limits^{{{\text{K}}_{5} }} {\text{R}}_{2} {\text{HNa}}\quad \left( {{\text{precipitate}}} \right)\;{\text{pK}}_{{5}} = - {9}.{35} $$

where: RH is oleic acid; R^−^ is oleate ion; R_2_H^−^ is acid-soap complex; R_2_HNa is acid-soap salt and R_2_^2−^ is oleate dimer, respectively.

**Figure 5:**
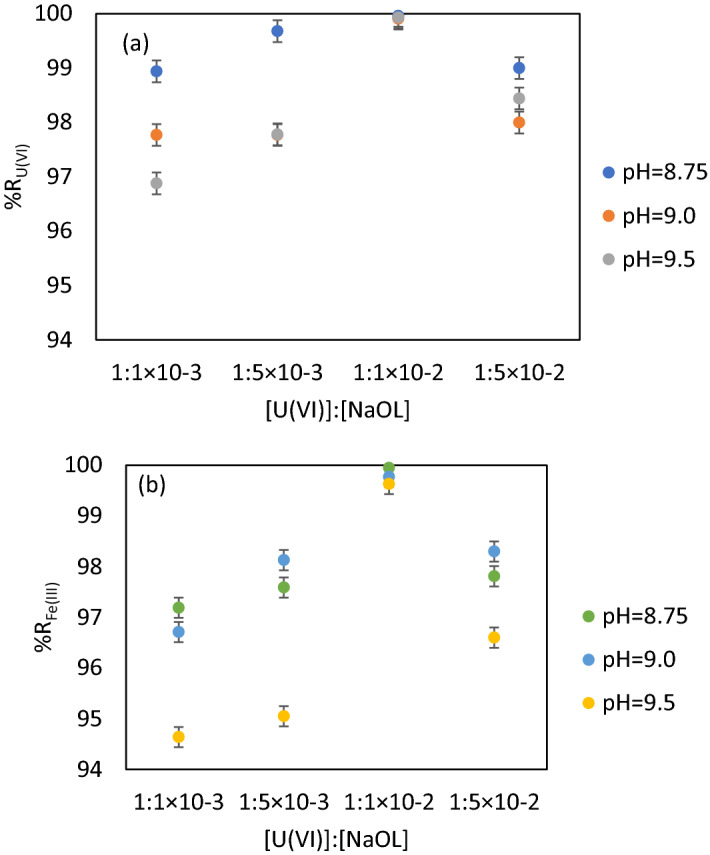
(**a**) %R_U(VI)_ = f([U(VI)] : [NaOL]) in the optimal pH range; (**b**) %R_Fe(III)_ = f([U(VI)] : [NaOL]) in the optimal pH range (V_sample_ = 200 mL, contact time 30 min., stirring rate 250 RPM, molar ratio [U(VI)] : [Fe (III)] = 1 : 75, p = 4 × 10^5^ N·m^−2^, dilution ratio V_sample_ : V_water_ = 3 : 1).

The results of the experiments are showed in Fig. 5.

The results shown in Fig. 5 suggest that the most reliable molar ratio is [U(VI)] : [NaOL] = 1 : 1 × 10^–2^.

#### Contact time U(VI) with Fe (III) and NaOL

**Figure 6:**
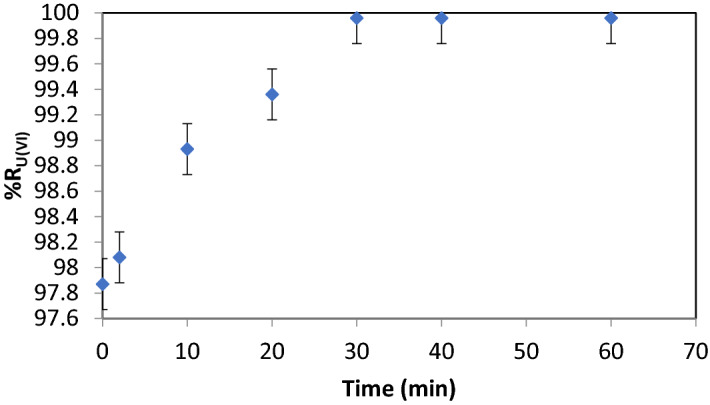
%R = f (contact time), V_sample_ = 200 mL, stirring rate 250 RPM, pH = 8.75, [U(VI)] : [Fe (III)] : [NaOL] = 1 : 75 : 1 × 10^–2^, p = 4 × 10^5^ N·m^−2^, dilution ratio V_sample_ : V_water_ = 3 : 1

The contact time includes both the time required to prepare the sorbent in situ and the time of pH adjustment; the determined working pH value of 8.75 was in accordance with the literature data^24,25,69^ regarding the formation of the Fe_2_O_3_ ∙ n H_2_O precipitate within the limits 7.0–9.5 as shown in Fig. 2. %R values as a function of contact time are shown in Fig. 6.

It can be observed that after 30 min the removal efficiency (%R) reaches the maximum value of 99.96. An additional increase in contact time determines no variation in removal efficiency (%R = 99.96). Therefore, the chosen contact time was 30 min because any other higher value it is not justified.

#### Stirring rate

This factor is important in the sorption stage of U(VI) on the sorbent. High stirring velocities determine smaller sizes of sorbent flake and the decrease of the U(VI) removal efficiency.

**Figure 7:**
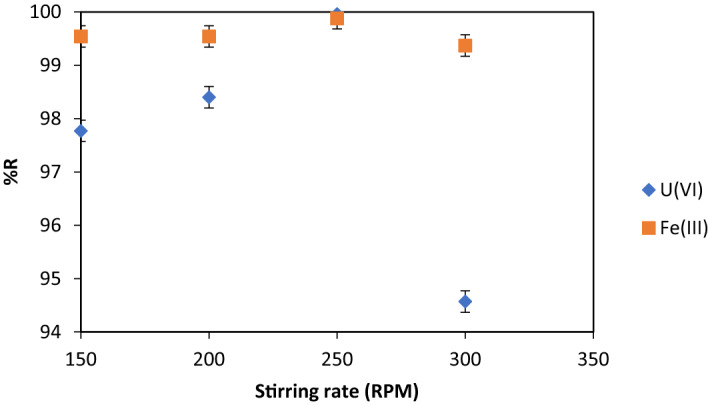
%R = f (stirring rate), V_sample_ = 200 mL, contact time 30 min, pH = 8.75, molar ratio [U(VI)] : [Fe (III)] : [NaOL] = 1 : 75 : 1 × 10^–2^, p = 4 × 10^5^ N·m^−2^, dilution ratio V_sample_ : V_water_ = 3 : 1

Figure 7 points out that 250 RPM is the best stirring rate to get U(VI) and Fe (III) removal efficiencies > 98%.

#### The air pressure (p) in the pressurized water recipient

The air pressure in the pressurized water recipient of flotation cell ensures the formation of homogeneous bubbles capable of taking up the solid sorbent loaded with U(VI) and to ensure sufficient ascending force for the loaded sorbent to concentrate on the top of the flotation cell column. Therefore, a low air pressure does not ensure these conditions and favours the reverse process of depositing the loaded sorbent at the bottom of the flotation cell column^48,49^. Higher air pressure values produce turbulence with a negative impact on the stability of aggregate bubble-loaded sorbent.

**Figure 8:**
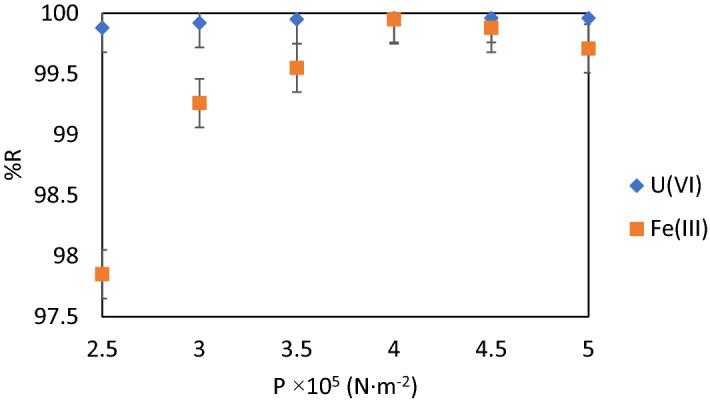
%R = f (p), V_sample_ = 200 mL, stirring rate 250 RPM, contact time 30 min, pH = 8.75, molar ratio [U(VI)] : [Fe (III)] : [NaOL] = 1 : 75 : 1 × 10^–2^, p = 4 × 10^5^ N·m^−2^, dilution ratio V_sample_ : V_water_ = 3 : 1

The results obtained and displayed in Fig. 8 suggests that the best working value of the air pressure is *p* = 4·10^5^ N·m^−2^, when the removal efficiency is maximum: %R_U(VI)_ = 99.96 and %R_Fe(III)_ = 99.95%, respectively.

#### The U(VI) concentration

The variation of the concentration of contaminants has an important impact on the separation efficiency because it determines the consumption of reagents and the volume of loaded sorbent.

As such, when the concentration reaches high values, it increases the weight of the loaded sorbent and decreases the floatability of solid phase.

**Figure 9:**
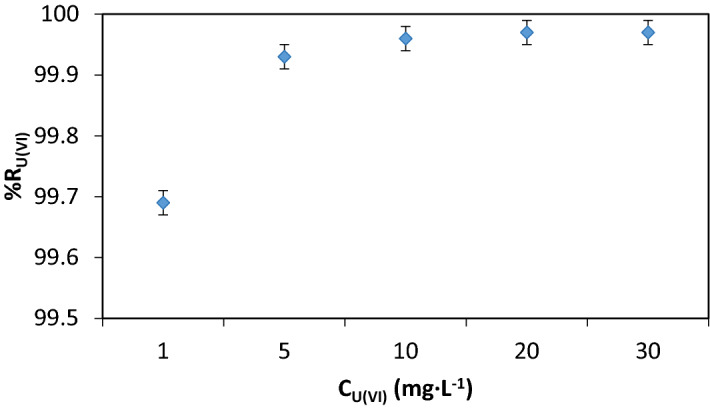
%R = f(U(VI)), V_sample_ = 200 mL, stirring rate 250 RPM, contact time 30 min, pH = 8.75, molar ratio [U(VI)] : [Fe (III)] : [NaOL] = 1 : 75 : 1 × 10^–2^, *p* = 4 × 10^5^ N·m^−2^, dilution ratio V_sample_ : V_water_ = 3 : 1

Figure 9 shows the effect of U(VI) concentration increase on the removal efficiency. Increases to 99.96% and then decreases slightly to concentrations greater than 20 mg·L^−1^.

### Optimum parameters

The optimal parameters (at maximum removal efficiency, %R) in order of the stages of the Sorption / Flotation process are:

- U(VI) concentration 10 mg L^−1^;
- Flotation pH range 7.5–9.5;
- U(VI) : sorbent dose, [U(VI)] : [Fe(III)] = 1: 75;
- Contact time U(VI) with Fe(III) = 25 min.;
- Stirring rate = 250 RPM;
- Molar ratio [U(VI)]: [NaOL] = 1: 1 × 10^–2^;
- Contact time U(VI) with Fe(III) and NaOL = 5 min.;
- Air pressure, *p* = 4 × 10^5^ N m^−2^;
- Flotation time = 5 min.

### The accompanying heavy metals ions’ interference

Seven samples (V_sample_ = 200 mL) were prepared in which U(VI), Cu (II), Cr (VI), and Mo (VI) were introduced 10 mg·L^−1^ each, were subjected to sorption / precipitate flotation under the optimal values of the previously established working parameters in order to observe the interactions between all ionic species. The results suggest that, in the multi-component solution, Cu(II) and Fe(III) precipitate, and U(VI) could be sorbed and/or precipitated. The Mo (VI) and Cr (VI) species can also be sorbed on Fe_2_O_3_ ∙ n H_2_O generated in situ.

In the case of Cu (II), the obtained results suggest that at working pH = 8.75 it precipitates as Cu(OH)_2_^49,70–72^.

The precipitates’ formation takes place in the order from the lowest to the most soluble product, i.e. Fe(OH)_3_ (K_sp_ = 2.79 × 10^–39^) < UO_2_(OH)_2_ (K_sp_ = 1.1 × 10^–20^) < UO_2_CO_3_ (K_sp_ = 1.8 × 10^–12^) < CuCO_3_ (K_sp_ = 1.4 × 10^–10^)^49,60,61,70–72^, according to the previously stated principle (3.1.1).

The main speciation of Cr(VI) at working pH = 8.75 is CrO_4_^2−^ according to the literature^73,74^.

In the case of Mo (VI) species, the researchers pointed out that the probable main speciation is MoO_4_^2−^ with a maximum concentration value at pH = 7, when the concentrations of the other two, H_2_MoO_4_ and HMoO_4_^−^, are very low^75^.

**Figure 10:**
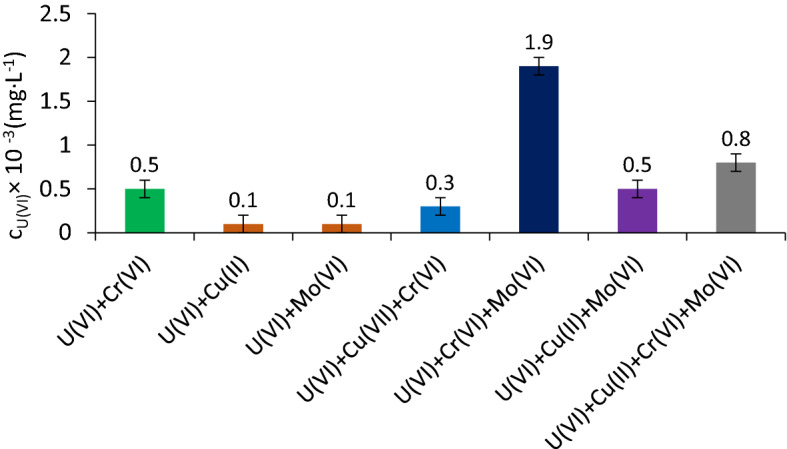
Influence of accompanying metallic ionic species C_i_ = 10 mg·L^−1^ on the variation of U(VI) content in the aqueous diluted systems after sorption / precipitate flotation.

Figure 10 shows the influence of the accompanying ions on U(VI) removal by sorption / precipitate flotation. It can be observed that, when Cu (II) and Mo (VI) species accompany U(VI) in bicomponent systems, the sorption U(VI) is not influenced by them unlike the case of Cr (VI), which decreases the removal efficiency of U(VI).

It can also be observed that following the sorption / precipitate flotation process, the removal efficiency of U(VI) from these studied aqueous systems is very high (%R > 99) considering that in solution the residual concentration of U(VI) has values in range 0.1–1.9 µg·L^−1^ which are much lower than the maximum permitted legal limit concentration (0.02 mg·L^−1^) stipulated by WHO regulations.

Other research studies presenting interactions in the aqueous species of U(VI) and the heavy metals accompanying of sorbent generated in situ have pointed out dominant metallic ionic specioation in the dilute aqueous systems, which are similar to those studied.

Riba et. al. has showed that for a solution with [U(VI)] = 4.2 mM (10 mg·L^−1^) in contact with 1.2% O_2(g)_ and 0.017% CO_2(g)_ for a pH range of 8 to 9 the dominant species are [UO_2_(CO_3_)_3_]^4−^ and [UO_2_(CO_3_)_2_]^2−^^25^.

Wanze et.al. has pointed out for [U(VI)] = 4.2 × 10^–6^ M dissolved in 0.01 M NaCl solution in the presence of carbonate [CO_3_^2−^] = 1 × 10^−2^ M there are the same dominant speciations^69^.

The presence of [MoO_4_^2−^] was demonstrated by Mitchell in the system with [Mo (VI)] = 0.3 and 1 mM (3 mg/L and 100 mg/L) at a pH range 2 to 7^75^.

According to Matis and Mavros in a diluted aqueous system containing Cu (II) = 10 mg/L at pH range 8 to10 precipitates Cu(OH)_2_^33^.

For [Cr (VI)] between 10^–4^ and 6 × 10^−4^ M in the pH range 1 to 12, the dominant speciation is CrO_4_^2−^^74,76^.

The results obtained demonstrate the presence of a competition between the metallic ion ionic species present in order to bind to the active surface of the sorbent charge with electric charge^74^. Since the zero sorption point of sorbent changes with increasing amount of Fe_2_O_3_ ∙ n H_2_O^64^, the obtained results suggest that Cu (II) species precipitate and Cr (VI) and Mo (VI) are removed from aqueous solution by sorption. The experimental results point out that it is possible that U(VI) is electrostatically bound to the electrically charged surface of the sorbent as a carbonate complex.

Experimental results prove that the accompanying heavy metals do not significantly influence the separation efficiency.

From the study of inflencing factors correlated with the maximum efficiency of U(VI) separation, it results that the optimal working parameters of U(VI) separation by sorption / precipitate flotation are: pH range 7.0–9.5, stirring rate 250 RPM, contact time 30 min, molar ratio [U(VI)] : [Fe(III)] : [NaOL] = 1 : 75 : 1 × 10^–2^, p = 4·10^5^ N·m^−2^, dilution ratio V_sample_ : V_water_ = 3 : 1, flotation time 5 min, depending on initial concentration range of U(VI) = 1–30 mg·L^−1^.

The optimum working conditions established for the synthetic aqueous systems were validated on real mine water samples and very good results have been obtained.

### The interaction of sorbent with U(VI) and the accompanying heavy metals

Preliminary data on the interaction between U(VI) and sorbent were obtained using the FT-IR spectra analysis of two samples obtained under optimal working conditions for sorption / precipitate flotation Sample 1—Fe_2_O_3_∙nH_2_O and Sample 2—Fe_2_O_3_∙nH_2_O with U(VI) carbonated complex.

Both spectra include the 3400 cm^−1^ IR band that can be assigned to the stretching modes of H_2_O molecules or the coating of hydrogen-bonded surface OH groups, while the 3037 cm^−1^ IR band is due to the presence of OH stretching mode in α-FeOOH and a corresponding prominent peak H_2_O coordinated or adsorbed close to 1620 cm^−1^^77^.

The U(VI) carbonate complex’s ions fixing on the adsorbent seems to be emphasized by the movement which is observed from 653 cm^−1^ to 626 cm^−1^ in Sample 2. The claim appears to be supported by the positive potential value near pH = 8.0 ^66^.

Table 1 presents the characteristic bands attributed to the sublates obtained after the U(VI) separation from Cr(VI), Cu(II), and Mo(VI) by sorption/precipitate flotation.

**Table 1:** Characteristic bands of sublates obtained after the separation by sorption/precipitate flotation of U(VI) from Cr (VI), Cu(II), and Mo(VI).

Characteristic bands, cm^−1^									Characteristic bands attribution	References
Sample *I*	Sample *A*	Sample *B*	Sample *C*	Sample *E*	Sample *D*	Sample *F*	Sample *G*	Sample *H*		
3609- 3305 3037	3609- 3305 3037	3609- 3305 3037	3679- 3218 3048	3692- 3246 3034 (s)	3685- 3212 3041	3685- 3233 3048	3685- 3233 3048	3678- 3240 3048 (s)	ν_OH non-assoc_ ν_OH assoc_ ν_HOH_	^78–81^
2854 2728	2854 2728	2854 2724	2857 2729	2850 2701	2850 2723 1964	2843 2715 2347	2843 2715 2347	2850 2701 2347	ν_CH_	^80^
1634	1620	1634	1632	1639	1639	1632	1639	1639	δ_OH_, δ_HOH_	^79,81^
1486*	1521	1542 1493*	1512 1335*	1518 1327*	1519 (s) 1328*	1540 (s) 1306*	1526 (s) 1328*	1512 1327*	${\nu }_{{CO}_{3}^{2-}}$ ${\nu }_{{COO}^{-}}^{*}$	^80,82^
1000–800	–	957 858	903	–	–	–	–	–	${\delta }_{{CO}_{3}^{2-}}$	^80,82^
^b^682 ^b^647 ^b^612	^a^703 626	^a^703 ^c^619	^d^683	^d^690	^f^669	^d^683	676	669	^a, b^ (ν_Fe-O_, δ_Fe-O_) ^c^Fe-CO in the same plane complex [(UO_2_)_2_(OH )_2_]^2+^ + CO_3_^2−^ ^f^ν_Cu-O_, δ_Cu-O_	^83–85^
569 534	556 527	534	506	541	556 506	542	542	563	ν_Fe-O_	^79,83,84^
499 463	478	485	414	470		428	421	470	δ_Fe-O_	^79,86–88^

Where : Sample *I* = Fe_2_O_3_·nH_2_O symbolized as Fe (III); Sample *A* = Fe(III) + U(VI); Sample *B* = Fe(III) + U(VI) + Cr(VI); Sample *C* = Fe(III) + U(VI) + Cu(II); Sample *E* = Fe(III) + U(VI) + Mo(VI); Sample *D* = Fe(III) + U(VI) + Cr(VI) + Cu(II); Sample *F* = Fe(III) + U(VI) + Cr(VI) + Mo(VI); Sample *G* = Fe(III) + U(VI) + Cu(II) + Mo(VI); Sample *H* = Fe(III) + U(VI) + Cr(VI) + Cu(II) + Mo(VI).

All FT-IR spectra with the characteristic bands shown in Table 1 present the following specific peaks:

- In the 3000–3650 cm^−1^ range are attributed to associated and non-associated hydroxyl groups;
- In the 1620–1634 cm^−1^ range attributed to the water adsorbed on the in situ generated Fe_2_O_3_·nH_2_O surface;
- The characteristic bands around 1500 cm^−1^ value attributed to the carbonate ions stretching vibration, which are present for *I* (Fe(III)) at 1486 cm^−1^ , for *A* (Fe(III) + U(VI)) at 1521 cm*-1*, for *B*(Fe(III) + U(VI) + Cr(VI)) at 1542 cm^−1^, for *C*(Fe(III) + U(VI) + Cu(II)) at 1512 cm^−1^, for *E*(Fe(III) + U(VI) + Mo(VI)) at 1518 cm^−1^, for *D* (Fe(III) + U(VI) + Cr(VI) + Cu (II)) with shoulder at 1519 cm^−1^, for *F*(Fe(III) + U(VI) + Cr(VI) + Mo(VI)) with shoulder at 1540 cm^−1^, for *G*(Fe(III) + U(VI) + Cu(II) + Mo(VI)) with shoulder at 1526 cm^−1^ and for *H*(Fe(III) + U(VI) + Cr(VI) + Cu(II) + Mo(VI)) at 1512 cm^−1^;
- The characteristic bands around 1400 cm^−1^ value may be attributed to the deformation vibration bond of FeOOH and they are present in all samples except sample *I* (Fe(III)) suggesting that U(VI), Cr(VI), and Mo(VI) might be bonded on the sorbent surface and that Cu(II) might be precipitated as copper carbonate at the working pH;
- The characteristic bands at 703 cm^−1^ attributed ν_Fe-O_ is present in *A* (Fe (III) + U(VI)) and *B* (Fe (III) + U(VI) + Cr (VI)) samples and seems to suggest the possibility of U(VI) bonding on the in situ generated sorbent;
- The characteristic bands at 682 cm^−1^, 647 cm^−1^, and 612 cm^−1^ attributed to δ_Fe-O_ from sample *I* (Fe (III)) seem to point out the available active sites’ existence for U(VI) and accompanying elements ions bonding;
- The band characteristic to the complex [(UO_2_)_2_(OH)^2^]^2+^ + CO_3_^2−^ appears only in the systems : *C*(Fe(III) + U(VI) + Cu(II)) at 683 cm^−1^; *E*(Fe(III) + U(VI) + Mo(VI)) at 690 cm^−1^ and *F*(Fe(III) + U(VI) + Cr(VI) + Mo(VI)) at 683 cm^−1^;
- The bands δ_***Fe***−***O***_ and ν_***Fe***−***O***_ are also shifted towards lower values indicating that chemisorption might be possible at this level as well.

The FT-IR spectra analysis suggests that there is a possibility for the [(UO_2_)_2_(OH)_2_]^2+^CO_3_^2−^ complex’s formation considering that the reaction kinetics is of pseudo-second-order involving the chemisorption. At the same time at the working pH, Cu (II) can precipitate, and Cr (VI) and Mo (VI) to be adsorbed on the Fe (III) oxyhydroxide ^50,74^.

Table 2 shows the sublates’ thermal analysis’ results obtained after the separation by sorption/precipitate flotation of U(VI) from Cr (VI), Cu (II), and Mo (VI).

**Table 2:** The thermal analysis of sublates obtained after the separation by sorption/precipitate flotation of U(VI) from Cr (VI), Cu (II), and Mo (VI).

Sample	DTA			
	Temperature domain (^o^C)	$\pm \Delta m$ (%)	Maximum temperature DTA (^o^C)	Reaction type
Fe_2_O_3_·nH_2_O is symbolized as Fe (III)	20–120	−5.65	97.1	Endo
	120–350	−8.07	304.1	Exo
	350–800	−2.27	620.9	Exo
	800–900	−1.54	–	–
Fe (III) + U(VI)	20–120	−5.09	100.7	Endo
	120–350	−6.34	277.2 341.3	Exo Exo
	350–900	−1.48	513.7	Exo
Fe (III) + U(VI) + Cr (VI)	20–120	−4.57	108.8	Endo
	120–350	−6.62	273.3 347.5	Exo Exo
	350–900	−1.38	598.7	Exo
Fe (III) + U(VI) + Cu (II)	20–120	−6.21	107.6	Endo
	120–350	−6.96	275.8 346.2	Exo Exo
	350–900	−1.99	593.9	Exo
Fe (III) + U(VI) + Mo (VI)	20–120	−6.17	102.3	Endo
	120–350	−6.14	273.0 340.2	Exo Exo
	350–900	−1.42	536.9	Exo
Fe (III) + U(VI) + Cr (VI) + Cu (II)	20–120	−5.81	109.3	Endo
	120–350	−6.80	278.0	Exo
	350–900	−2.06	533.8	Exo
Fe (III) + U(VI) + Cr (VI) + Mo (VI)	20–120	−5.51	103.7	Endo
	120–350	−6.27	281.0 344.7	Exo Exo
	350–900	−1.35	555.3	Exo
Fe (III) + U(VI) + Cu (II) + Mo (VI)	20–120	−5.36	105.7	Endo
	120–350	−6.74	277.1 349.0	Exo Exo
	350–900	−1.86	586.2	Exo
Fe(III) + U(VI) + Cr(VI) + Cu(II) + Mo(VI)	20–120	−6.22	103.8	Endo
	120–350	−6.57	276.9 342.9	Exo Exo
	350–900	−1.55	549.2	Exo

The analysis of TG/DTG/DTA curves shows the sublates’ non-iso-thermal degrading process in the air atmosphere in the case of the bi-, three- and tetra component systems. The samples were subjected to three successive decomposing and water loss processes (Table 2).

The first endothermal process (20–120 °C) points out moisture’s complete loss. The analysed samples present similar moisture. The weight losses in this stage are about Δm_1_ = 4.57–6.21% at the maximum temperatures within the range 97.1–109.3 °C.

The samples seem to be stable within the temperature range of 120–250 °C. Then the second decomposition process follows, which is exothermal (250–350 °C) and represents the main degrading stage with the weight loss Δm_2_ = 6.14–8.07% at the maximum temperatures within the ranges 273.0–281.0 °C and 304.1–347.5 °C, respectively.

At higher temperatures (350–900 °C) the last exothermal process of thermal-oxidative decomposition of non-volatile products was obtained in the second degrading stage.

In all cases for the temperatures ranging within 513.7–620.9 °C the similar residual weights Δm_3_ = 1.35–2.06% point out the studied metallic ions’ oxides’ mixtures’ occurrence.

### Reproducibility and optimal parameters validation on real mine water samples

#### Reproducibility of U(VI) removal by sorption/precipitate flotation

Previously determined sorption/precipitate flotation technique optimal parameters were examined on 10 identical sample solutions (C_o_ = 10 mg/L) corresponding to two different molar ratios ([U(VI)] : [Fe (III)] : [NaOL] = 1 : 75 : 1 × 10^–2^ and 1 : 100 : 1 × 10^–2^), respectively, to calculate the U(VI) removal reproducibility by Student method (Table 3).

**Table 3:** Reproducibility of U(VI) removal by sorption/precipitate flotation.

No	Floated sample characteristics	C_t_, (mg·L^−1^)	Statistical probability
1	C_o_ = 10 mg·L^−1^ V_sample_ = 200 mL pH = 8.75 [U(VI)] : [Fe (III)] : [NaOL] = 1 : 100 : 1 × 10^–2^ V_sample_ : V_water_ = 3 : 1 *p* = 4·10^5^ N·m^−2^	0.01	$\overline{X }$= 0.008 S = 2.6667·10^–6^ ${S}_{\overline{X} }$ = 8.4328·10^–6^ P = 95% t·${S}_{\overline{X} }$ = 0.000018 C_t_ = 0.008 ± 0.000018 P = 99% t·${S}_{\overline{X} }$= 0.000032 C_t_ = 0.008 ± 0.000032
2		0.009	
3		0.008	
4		0.005	
5		0.007	
6		0.008	
7		0.006	
8		0.01	
9		0.009	
10		0.008	
11	C_o_ = 10 mg·L^−1^ V_sample_ = 200 mL pH = 8.75 [U(VI)] : [Fe (III)] : [NaOL] = 1 : 75 : 1 × 10^–2^ V_sample_ : V_water_ = 3 : 1 *p* = 4·10^5^ N·m^−2^	0.0044	$\overline{X }$= 0.00426 *S* = 2.593·10^–4^ ${S}_{\overline{X} }$ = 8.19998·10^–5^ *P* = 95% t·${S}_{\overline{X} }$= 0.00018285 C_t_ = 0.00426 ± 0.00018285 *P* = 99% t·${S}_{\overline{X} }$= 0.000259939 C_t_ = 0.00426 ± 0.000259939
12		0.0043	
13		0.0040	
14		0.0044	
15		0.0039	
16		0.0041	
17		0.0045	
18		0.0040	
19		0.0046	
20		0.0044	

$\overline{X }$ mean of the samples.

*S* standard deviation of one measurement.

${S}_{\overline{X} }$ standard deviation of the mean.

*C*_*t*_ U(VI) concentration after flotation.

*P* probability that C_t_ be within a range of values.

*t* t (Student) variable.

#### Optimal parameters validation on real mine water samples

The mine water samples (MW1-MW3) were collected from a former uranium mining site in the Banat region and their chemical composition is shown in Table 4. They were processed according to the proposed flowsheet (Fig. 12) with and without pH adjustment respectively. The pH was adjusted with 0.1 M HCl solution to the working value of 8.75.

**Table 4:** Chemical composition of real water samples (mg·L^−1^).

Sample	pH at 22 °C	U	Mo	Sn	Zn	Pb	Cr (VI)	Co	Cu	Ni	Na_2_CO_3_	NaHCO_3_
MW1	9.43	13.8	0.264	< 0.001	0.01	< 0.001	< 0.001	0.01	< 0.001	< 0.001	642.3	1527.1
MW2	9.59	10.35	0.284	< 0.001	< 0.001	< 0.001	< 0.001	0.01	< 0.001	< 0.001	749.4	1527.1
MW3	9.64	16.40	0.270	< 0.001	< 0.001	< 0.001	0.01	< 0.001	< 0.001	< 0.001	642.3	1527.1

**Figure 11:**
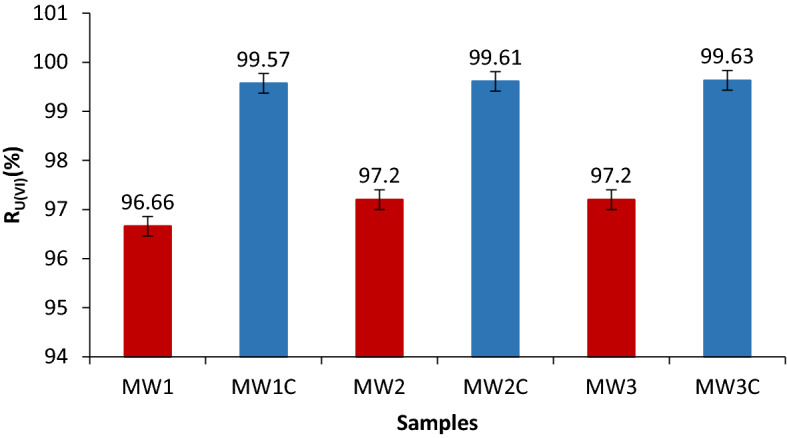
%R_U(VI)_ = f (pH adjustment), V_sample_ = 200 mL, stirring rate 250 RPM, contact time 30 min, molar ratio [U(VI)] : [Fe (III)] : [NaOL] = 1 : 75 : 1 × 10^–2^, p = 4 × 10^5^ N·m^−2^, dilution ratio V_sample_ : V_water_ = 3 : 1, where  MW1–MW3—samples without pH adjustment and  MW1C–MW3C—samples with pH adjustment.

It was observed that the U(VI) removal efficiency was higher after pH adjustment, so that the sorption flotation was very efficient (Fig. 11).

**Figure 12:**
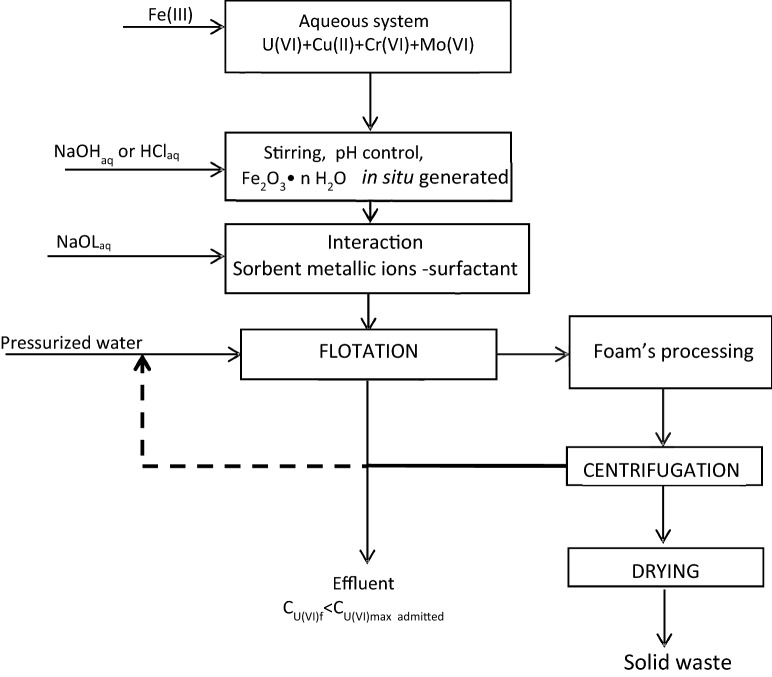
The separation scheme for the treatment of a multi-component system by sorption flotation adapted to the studied system^51^.

Figure 12 summarizes a proposed technological processing diagram (flowsheet) of the multi-contaminated aqueous system by sorption flotation.

**Figure 13:**
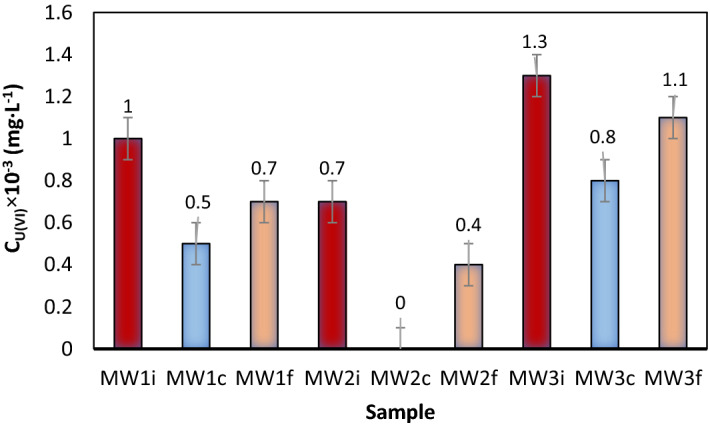
U(VI) residual concentration change in three real water samples after immobilization—sorption / flotation processing, where:  MW1i–MW3i is the liquid phase resulting after immobilization on NMS;  MW1c–MW3c is a liquid phase with pH adjusted with FeCl_3_ 0.1 ;  MW1f.–MW3f. is the liquid phase resulting after the immobilization on NMS, decantation, collector addition, and flotation.

In case the samples were processed without pH adjustment the separation efficiencies were 96.6% for sample MW1 and 97.2 for MW2 and MW3 samples, respectively (Fig. 13).

In case the samples were processed with pH adjustment at pH = 8.75 with 0.1 M HCl solution %R_U(VI)_ > 99 was obtained for MW1C–MW3C samples (Fig. 13).

One can note that U(VI) removal efficiency was higher for the pH-adjusted samples than for the others, confirming the optimal values of the previously studied parameters.

The generated solid waste may be stored or recycled as a U(VI) secondary source for the manufacture of nuclear fuel.

**Figure 14:**
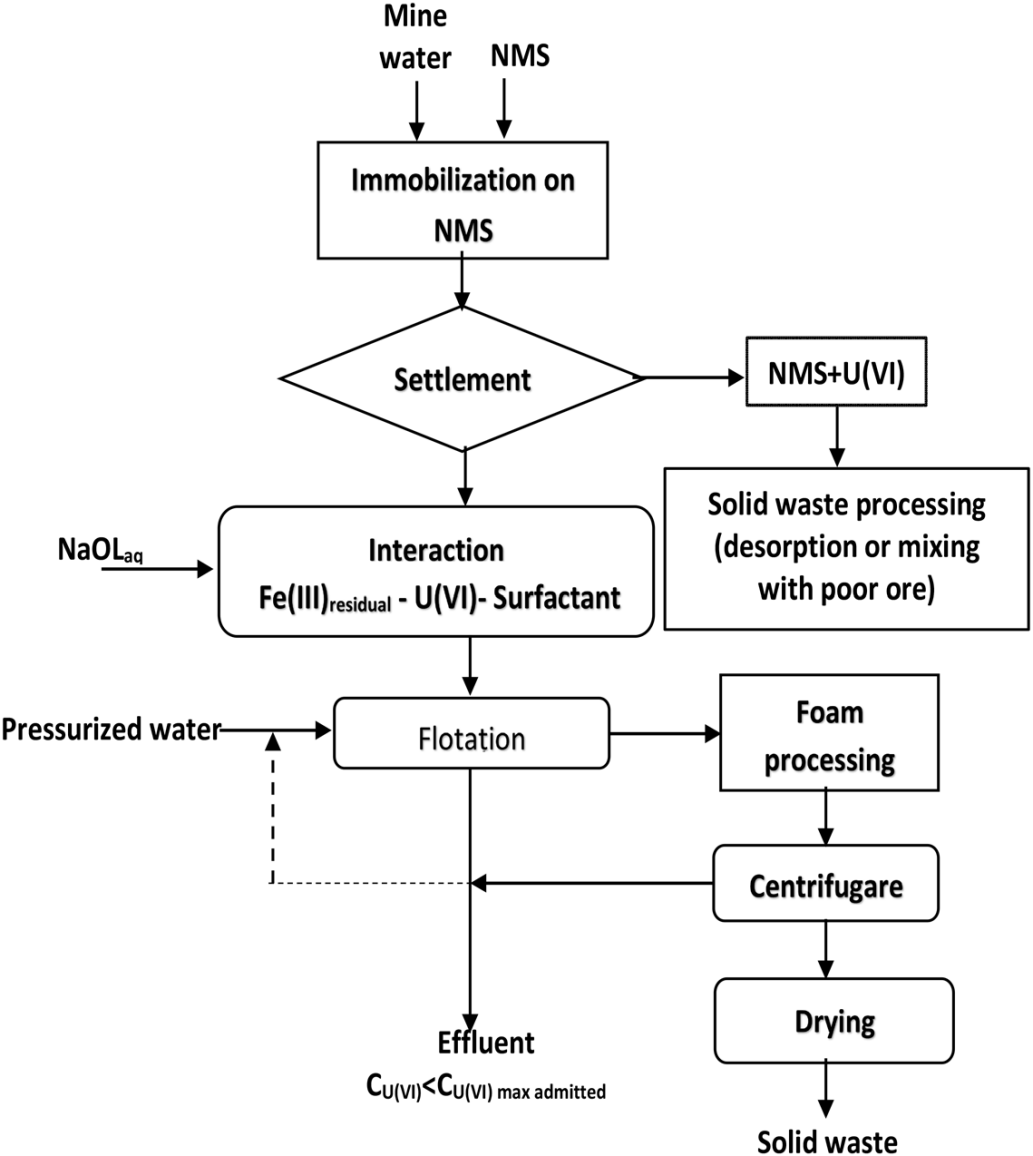
Immobilization and flotation without pH adjustment and in situ generated Fe_2_O_3_·nH_2_O using only the Fe (III)_residual_ after U(VI) immobilization on NMS.

**Figure 15:**
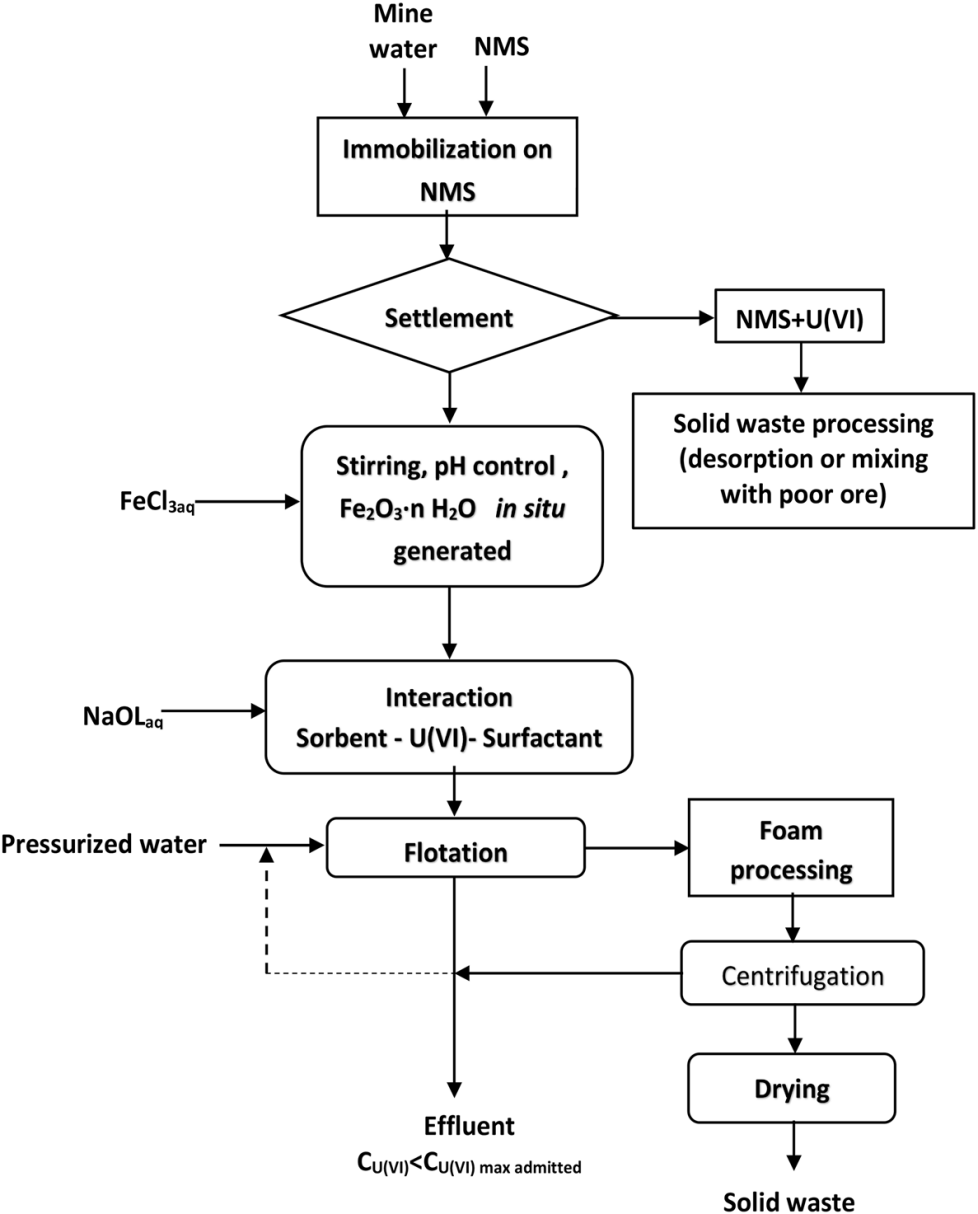
Immobilization and flotation where for pH adjustment 0.1 M FeCl_3_ solution is used, which is the reagent for sorbent in situ generation.

The optimal parameters validation of tandem process immobilization on NMS-flotation on real water samples was performed in two variants:

- **Without pH adjustment and sorbent addition:** The real water samples with the chemical composition shown in Table 4 (300 mL) MW1–MW3 were pre-treated with 0.15 g NMS and were contacted for 30 min under 250 RPM stirring. The solid phase was separated by decantation. To the resulting liquid phase, MW1i–MW3i, the appropriate amount of 0.25 × 10^−3^ M NaOL solution was added and flotated without pH adjustment and without addition of FeCl_3_ 0.1 M because the Fe^2+^ and Fe^3+^ supplied by the NMS in the filtered solution was used as an adsorption support. After flotation, the water samples MW1f.–MW3f. were obtained (Fig. 14).
- **With pH adjustment and sorbent addition:** The real water samples with the chemical composition shown in Table 4 (300 mL) MW1–MW3 were pre-treated with 0.15 g NMS for 30 min under 250 RPM stirring. The solid phase was separated by decantation. To the resulting liquid phase, MW1i–MW3i, the pH was adjusted using 0.1 M FeCl_3_ solution to avoid the addition of the foreign ion, the appropriate amount of 0.25 × 10^−3^ M NaOL solution was added and after flotation samples, MW1c–MW3c were obtained (Fig. 15).

Figure 13 shows the U(VI) residual content after immobilization and flotation of real water samples.

Two separation schemes’ versions, which use both U(VI) removal methods, have resulted as follows: one without pH adjustment and without in situ generation of Fe_2_O_3_·nH_2_O (Fig. 14) and another one with pH adjustment and with in situ Fe_2_O_3_·nH_2_O generated (Fig. 15).

The obtained results on the real water samples suggest that U(VI) separation by sorption/precipitate flotation may be used either as a single method or as an additional stage in the case when Fe^0^-based nanomaterials are used in situ.

## Conclusions

This paper studied the possibility of removing U(VI) and some associated metallic ions, specific to multicomponent aqueous systems in the uranium industry, by an efficient removal process as sorption on sorbent generated in situ (Fe_2_O_3_ ∙ nH_2_O) followed by flotation (%R_U(VI)_ and %R_Fe(III)_ > 99) in working conditions (C_i,U(VI)_ = 10 mg ∙ L^−1^, pH range = 7.5–9.5, [U(VI)] : [Fe(III)] = 1 :75, contact time = 30 min., stirring rate = 250 RPM, [U(VI)] : [NaOL] = 1 : 1 × 10^–2^, p = 4 × 10^5^ N ∙ m^−2^, flotation time = 5 min.).

In establishing the separation process, the existing speciations, possible interactions and probable species participating in the process (pH range 7.0–9.5) were taken into account: U(VI) hydroxide complex (UO_2_(OH)_2_, [UO_2_(OH)_3_]^−^ and [(UO_2_)_3_(OH)_7_]^−^), U(VI) carbonate complexes (UO_2_CO_3_, [UO_2_(CO_3_)_2_]^−2^, [UO_2_(CO_3_)_3_]^−4^ and [(UO_2_)_2_CO_3_(OH)_3_]^−^), Fe(III) hydroxide complex (Fe_2_O_3_ ∙ nH_2_O).

To explain the separation mechanism were registered: FTIR spectra (range 400–4000 cm^−1^) and derivatograms (range 20–1000 °C) of the solid loaded with U(VI) concentrated in foam (sublate). Thus FT-IR analysis has pointed out the possibility of forming the complex [(UO_2_)_2_(OH)_2_]^2+^CO_3_^2−^, which may be bond to Fe (III) oxyhydroxides formed at upon immobilization on NMS and (in case of the flotation process in tandem by pre-treated of aqueous sistems with the immobilization on Fe-based nanomaterials) / or generated in situ Fe_2_O_3_·n H_2_O sorbent formed in precipitate flotation process, as well.

In the case of applying the proposed procedure on real samples pre-treated with NMS (Fe^0^ based nanomaterial), without pH adjustment and FeCl_3_ addition, the solid phase loaded with metallic ions was separated by decantation. The separation efficiency was more than 99%.

In the case of applying the proposed procedure on real samples pre-treated with NMS (Fe^0^ based nanomaterial), with pH adjustment and FeCl_3_ addition, the solid phase loaded with metallic ions was separated by decantation. The pH of the aqueous phase after settling is adjusted to pH = 8.75 by adding FeCl_3_ solution. The adsorbent is generated in situ, it will be loaded with metallic ions remaining from their immobilization on the NMS and finally flotation stage is applied. The separation efficiency was more than 99%.

Validation of optimal parameters on multicomponent real mine water samples and the ability of Fe_2_O_3_ ∙ nH_2_O to interact with multiple metallic speciations concludes that sorption/precipitate flotation tandem could be considered an advantage complementary in remediation technology, as a novelty in this area.

## Data Availability

The datasets used and/or analyzed during the current study are available from the corresponding author upon reasonable request.
